# Analysis of the temporal changes in management of early-stage cervical cancer over a 10-year period in Germany: a multicenter retrospective analysis of the JAGO/NOGGO

**DOI:** 10.3389/fonc.2026.1916573

**Published:** 2026-08-05

**Authors:** Lena Steinkasserer, Lisa Marie Wilhelm, Julie Schambach, Henning Schäffler, Morva Tahmasbi Rad, Fabienne Schochter, Davit Bokhua, Zoe Huber, Peter Hillemanns, Hermann Hertel, Chiara Flethe, Klaus Pietzner, Jalid Sehouli

**Affiliations:** 1 Department of Gynecology and Obstetrics, Hannover Medical School, Hannover, Germany; 2 Clinic for Gynecology and Obstetrics, University Hospital Frankfurt am Main, Frankfurt am Main, Germany; 3 Department of Gynecology and Obstetrics, University Hospital Jena, Jena, Germany; 4 Department of Gynecology and Obstetrics, University Hospital Ulm, Ulm, Germany; 5 Department of Gynecology, Center for Oncological Surgery, Campus Virchow Klinikum, Charite-Universitätsmedizin Berlin, Corporate Member of Freie Universität Berlin, Humboldt-Universität zu Berlin, Berlin Institute of Health, Berlin, Germany; 6 Young Academy of Gynecologic Oncology (JAGO), North-Eastern German Society of Gynecologic Oncology (NOGGO), Berlin, Germany

**Keywords:** early-stage cervical cancer, fertility-sparing surgery, hysterectomy techniques, minimally invasive surgery, real-world data, surgical management

## Abstract

**Background:**

Surgical management of early-stage cervical cancer has evolved substantially over the past decade. While the LACC trial raised concerns regarding minimally invasive radical hysterectomy, real-world practice continues to include radical, non-radical, and fertility-sparing approaches. This study evaluated operative strategies and temporal trends in Germany, particularly regarding the impact of the LACC trial.

**Methods:**

This retrospective multicenter study included 391 patients with early-stage cervical cancer (≤pT1, pN0, FIGO < IB1) treated at four German university hospitals between 2014 and 2024. Demographic, surgical, perioperative, oncologic, and follow-up data were analyzed descriptively, focusing on temporal changes in surgical management.

**Results:**

The median age at diagnosis was 44 years, and 95.9% of patients had an ECOG performance status of 0–1. Pathological staging revealed pT1b1 tumors in 56.0% of cases, while microinvasive disease (pT1a1–a2) accounted for 35.3%. Laparoscopic approaches declined from 66.5% before 2019 to 50.0% thereafter following publication of the LACC trial in 2018 (p < 0.001), with a corresponding increase in open surgery. Fertility-sparing procedures remained stable (conization 11.3%, trachelectomy 7.4%). Perioperative morbidity was low, with no postoperative complications in 84.4% of patients. Disease recurrence occurred in 5.9% of patients.

**Conclusions:**

This multicenter study provides a comprehensive overview of contemporary surgical management of early-stage cervical cancer in Germany. Surgical practice changed substantially over time, likely reflecting the combined influence of emerging clinical evidence, evolving guideline recommendations, and changing treatment paradigms; however, no uniform operative standard has emerged. Favorable perioperative and oncologic outcomes across approaches highlight the importance of patient selection and surgical expertise in contemporary cervical cancer care.

## Introduction

Cervical cancer remains one of the most common gynecologic malignancies worldwide, despite substantial advances in prevention and early detection. Due to the nationwide screening program in Germany early-stage disease accounts for a significant proportion of newly diagnosed cases. In 2022, 4388 patients with cervical carcinoma were diagnosed in Germany (1). A substantial proportion of these cases were early-stage tumors, with early cervical cancers (FIGO stage T1a and T1b) accounting for 45.2% of cases reported in 2021 (2). Early-stage cervical cancer is typically defined as tumors confined to the cervix, including FIGO stages IA to IB1, and offers a favorable prognosis when appropriately managed (3). For patients with early-stage cervical cancer, multiple therapeutic strategies are available, including surgical approaches, and increasingly individualized treatments tailored to tumor biology, fertility preservation, and comorbidities (3, 4).

Surgical treatment has undergone significant evolution in recent years. Historically, radical hysterectomy via laparotomy was the standard of care. However, minimally invasive surgery gained popularity due to its perceived benefits in terms of perioperative morbidity and recovery. This paradigm was challenged by the LACC trial, published in 2018, which reported inferior oncologic outcomes for minimally invasive radical hysterectomy compared to open surgery (5). Ramirez et al. followed up the patients for 4.5 years, showing an overall survival of 90.6% in the minimally invasive surgery group compared to 96.2% in the open surgery group (hazard ratio for death from any cause, 2.71; 95% CI, 1.32–5.59; P = .007). In light of the higher recurrence rates and inferior overall survival associated with minimally invasive procedures, open surgery was therefore considered the standard of care from 2019 onwoards (4, 6). Following the publication of the LACC trial, several large registry-based studies have compared open and minimally invasive surgical approaches, reporting heterogeneous oncologic outcomes and contributing to ongoing uncertainty regarding the optimal surgical strategy. The SHAPE trial demonstrated non-inferior pelvic recurrence rates after simple hysterectomy compared with radical hysterectomy in selected patients with low-risk early-stage cervical cancer. With a median follow-up of 4.5 years, the 3-year incidence of pelvic recurrence was 2.17% after radical hysterectomy and 2.52% after simple hysterectomy. In addition, simple hysterectomy was associated with significantly lower postoperative morbidity, particularly regarding urinary dysfunction, resulting in improved quality of life for affected patients (7).

Current international and national guidelines reflect this evolving evidence landscape. While they have largely adopted the findings of the LACC trial and recommend an open surgical approach for radical hysterectomy, uncertainty remains regarding the optimal extent of surgery and the role of minimally invasive techniques in selected patient populations. In addition, it is likely that surgical practice continues to vary between centers, with some institutions still performing minimally invasive procedures in carefully selected cases. Against this background, it remains important to understand how early-stage cervical cancer is currently managed in routine clinical practice.

In many other gynecological cancer entities, a clear trend toward surgical de-escalation has emerged, for example the approach to axillary dissection in breast cancer – now favoring procedures with reduced radicality and complication rates while maintaining oncologic outcomes (8). Whether a de-escalating strategy is applicable to early-stage cervical cancer remains an open and controversial issue. In this context, it is crucial to understand what constitutes the current standard of care in clinical practice, particularly in countries like Germany. Despite growing evidence, it remains unclear whether a consensus has been reached among gynecologic oncology centers or if practice patterns continue to vary widely in the face of clinical uncertainty. The primary objective of this multicenter study was to comprehensively evaluate the current management practices for early-stage cervical cancer in Germany during the period from 2014 to 2024, with a specific focus on the diagnostic strategies and surgical techniques employed. Secondary endpoints encompassed recurrence-free survival as well as overall survival, providing insight into the long-term oncologic outcomes associated with the various treatment modalities.

## Methods

### Patients

#### Inclusion criteria

Patients diagnosed with cervical cancer were identified, and those with early-stage disease were included in this retrospective observational study. For temporal analyses, patients were compared across predefined study periods. The year 2019 was selected *a priori* to evaluate changes in surgical management following publication of the LACC trial, whereas 2022 was analyzed separately to assess practice patterns after formal incorporation of these findings into the German S3 guideline. These analyses were intended to distinguish between the potential influence of emerging clinical evidence and subsequent guideline implementation. The study followed a multicenter approach, conducted at university hospitals in Frankfurt, Hannover, Jena, and Ulm, Germany. The observation period spanned from June 2014 to June 2024.

The study was approved by the Ethics Committees of Goethe University Frankfurt (No. 2024-1809), and the university hospitals of Hannover (No. 11462_BO_K_2024), Jena (No. 2024-3450-Daten), and Ulm (No. 275/24).

### Data collection

Medical records of patients with early-stage cervical cancer were reviewed. Eligible cases were defined as having tumor stage <T2, lymph node status N0, and FIGO stage ≤IB1. Data collected included demographic information, age at disease onset, presence of precancerous lesions, HPV status, staging, surgical methods, and postoperative treatments. Missing data were retained in the dataset and reported as not available (N.A.). Analyses were performed using available-case data for the respective variables. Follow-up duration was calculated from the date of initial diagnosis to the date of last documented clinical contact or death, whichever occurred last. Follow-up assessments were generally conducted in accordance with contemporary German S3 guideline recommendations for cervical cancer and local institutional practice at the participating centers.

### Statistical analysis

Statistical analyses were performed using Graph Pad Prism 9 (GraphPad Software, La Jolla, CA). Continuous variables were assessed for normality using the Shapiro–Wilk test. Normally distributed variables are presented as mean ± standard deviation and were compared using Student’s t-test or one-way ANOVA, as appropriate. Categorical variables were compared using the chi-square test or Fisher’s exact test. Missing data were retained in the dataset and reported as not available (N.A.). Analyses were performed using available-case data for the respective variables.

All statistical tests were two-sided, and a p-value < 0.05 was considered statistically significant.

## Results

### Study population

**Figure 1:**
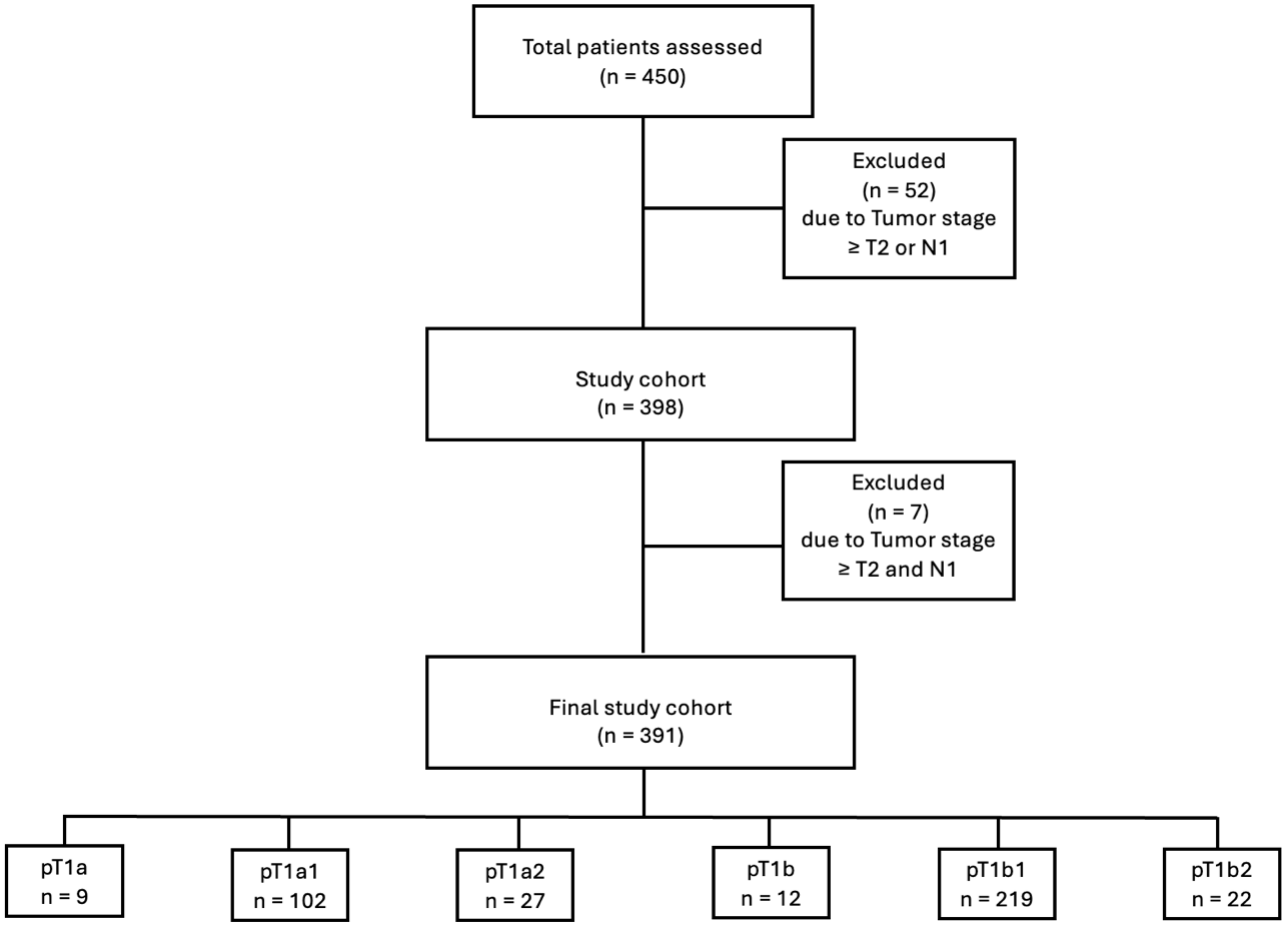
Flowchart of patient selection and study cohort composition. A total of 450 patients were screened for eligibility. After exclusion of patients with tumor stage ≥ T2 or nodal involvement (N1), 391 patients remained in the final study cohort. The distribution of pathological tumor stages (pT) in the final cohort is displayed.

Between 2014 and 2024, a total of 391 patients with histologically confirmed early-stage cervical cancer (≤pT1, pN0) were included in this multicenter real-world analysis (**Figure 1**). The median age at diagnosis was 44 years (IQR 36–53). Most patients presented with a good performance status (ECOG 0–1 in 95.9%), reflecting a cohort suitable for surgical treatment. Pathological tumor staging confirmed early disease in all patients. 56.0% of tumors were classified as pT1b1, followed by microinvasive disease (pT1a–1a2, 35.3%), representing stages in which the extent and surgical approach are particularly debated in the context of the LACC trial (**Table 1**).

**Table 1:** Study population.

Variable	n (%) or median (IQR)
Age at diagnosis (years)	44 (36–53)
ECOG performance status	
ECOG 0	322 (82.4)
ECOG 1	53 (13.6)
ECOG 2	8 (2.0)
Not available	8 (2.0)
Pathological tumor stage (pT)	
pT1a	9 (2.3)
pT1a1	102 (26.1)
pT1a2	27 (6.9)
pT1b	12 (3.1)
pT1b1	219 (56.0)
pT1b2	22 (5.6)
Pathological lymph node status	
pN0	391 (100)
Histological subtype	
Squamous cell carcinoma	278 (71.1)
Adenocarcinoma	100 (25.6)
Neuroendocrine carcinoma	2 (0.5)
Other	9 (2.3)
Not available	2 (0.5)

Eligible cases were defined as patients with early-stage cervical cancer based on pathological tumor stage pT1 and node-negative disease (pN0). FIGO stage was recorded where available. However, because the study period spanned the introduction of the revised FIGO 2018 classification, FIGO stage was not used as the primary inclusion criterion to ensure comparability across the entire cohort. Instead, eligibility was based on tumor size and surgical treatment characteristics, and tumors up to 4 cm in maximum diameter were therefore included.

### Surgical management

A wide range of surgical approaches were applied for the treatment of early-stage cervical cancer. When considering the entire study period without temporal stratification, laparoscopic radical hysterectomy (LRH) was the most frequently performed radical procedure and was carried out in 98 patients (25.1%), followed by abdominal radical hysterectomy (ARH) in 74 patients (18.9%). Vaginally assisted laparoscopic radical hysterectomy (VALRH) was performed in 43 patients (11.0%), while laparoscopic-assisted vaginal radical hysterectomy (LAVRH) was documented in 8 patients (2.1%). Total mesometrial resection (TMMR) was documented in the surgical reports of 17 patients (4.4%).

Among non-radical hysterectomy procedures, total laparoscopic hysterectomy (TLH) was performed in 46 patients (11.8%), and laparoscopic-assisted vaginal hysterectomy (LAVH) in 30 patients (7.7%). Abdominal simple hysterectomy was rarely used (2 patients, 0.5%), and no patients underwent primary vaginal hysterectomy. Fertility-sparing surgical procedures represented a relevant proportion of cases. Cervical conization was performed in 44 patients (11.3%), and trachelectomy in 29 patients (7.4%). Fertility preservation was attempted in 73 patients (18.7%), corresponding to all patients who underwent fertility-sparing procedures, including cervical conization (n = 44) and trachelectomy (n = 29). Fertility preservation was successfully achieved in 65 patients (16.6%). A history of conization was documented in 203 patients (51.9%), whereas 150 patients (38.4%) had no documented prior conization. Information regarding conization status was unavailable in 38 patients (9.7%). Regarding concomitant surgical procedures, salpingectomy was performed in 245 patients (62.7%), and adnexectomy in 142 patients (36.3%). Ovariopexy was performed in 39 patients (10.0%). A uterine manipulator was used in 84 procedures (21.5%), whereas most surgeries were performed without manipulator use. The most used uterine manipulator was the “Hohl” uterine manipulator. Intraoperative frozen section analysis of lymph nodes was performed in 212 patients (54.2%) (**Table 2**). Lymph node staging was most frequently performed using sentinel lymph node biopsy, which was applied in 194 patients (49.6%), followed by systematic laparoscopic lymphadenectomy in 99 patients (25.3%). Imaging-based lymph node assessment using CT (14 patients, 3.6%) or MRI (11 patients, 2.8%) was uncommon, while the lymph node staging modality was not documented in 73 patients (18.7%). Regarding the sentinel lymph node technique, gamma probe detection was the most frequently used method (149 patients, 38.1%), followed by indocyanine green (ICG) fluorescence in 127 patients (32.5%) and blue dye mapping in 117 patients (29.9%). In many cases, more than one sentinel mapping technique was applied in combination. In 144 patients (36.7%), more than one sentinel mapping technique was applied in combination. All results are shown in **Table 2**.

**Table 2:** Surgical management.

Surgical procedure	n (%)
Radical hysterectomy	
Laparoscopic radical hysterectomy (LRH)	98 (25.1)
Abdominal radical hysterectomy (ARH)	74 (18.9)
Vaginally assisted laparoscopic radical hysterectomy (VALRH)	43 (11.0)
Laparoscopic-assisted vaginal radical hysterectomy (LAVRH)	8 (2.1)
Non-radical hysterectomy	
Total laparoscopic hysterectomy (TLH)	46 (11.8)
Laparoscopic-assisted vaginal hysterectomy (LAVH)	30 (7.7)
Abdominal simple hysterectomy	2 (0.5)
Total mesometrial resection (TMMR)	17 (4.4)
Fertility-sparing surgery	
Cervical conization	44 (11.3)
Trachelectomy	29 (7.4)
Fertility preservation	
Fertility preservation attempted	73 (18.7)
Fertility preservation achieved	65 (16.6)
Concomitant surgical procedures	
Salpingectomy	245 (62.7)
Adnexectomy	142 (36.3)
Frozen section of lymph nodes	212 (54.2)
Ovariopexy	39 (10.0)
Lymph node staging modality	
Laparoscopic sentinel lymphadenectomy	194 (49.6)
Laparoscopic systematic lymphadenectomy	99 (25.3)
CT-based nodal staging	14 (3.6)
MRI-based nodal staging	11 (2.8)
Not documented	73 (18.7)
Sentinel lymph node mapping technique*	
Gamma probe	149 (38.1)
Indocyanine green (ICG)	127 (32.5)
Blue dye	117 (29.9)

*In some cases, more than one sentinel mapping technique was applied in combination.

### Subsequent surgical intervention and ovarian preservation

Subsequent surgical intervention following the initial operation were rarely required. LRH was performed as completion surgery in 3 patients (0.8%), ARH in 4 patients (1.0%), VH and LAVH in each 1 patient (0.3%).

### Operative parameters and perioperative outcomes

Operative parameters and perioperative outcomes are summarized in **Table 3**. Operative parameters differed substantially depending on the type of surgical procedure. Median operative time was longest for radical hysterectomy (233 minutes, IQR 157–335), compared with non-radical hysterectomy (97 minutes, IQR 81–154) and fertility-sparing procedures (30 minutes, IQR 11.5–140). The fertility-sparing category comprised both cervical conization and trachelectomy; the relatively short median operative time reflects the substantial proportion of conization procedures within this group.

**Table 3:** Operative parameters and perioperative outcomes.

Variable	Radical hysterectomy	Non-radical hysterectomy	Fertility-sparing procedures
	(n, %) or median (IQR)	(n, %) or median (IQR)	(n, %) or median (IQR)
Operative time (minutes)	233 (157–335)	97 (81–154)	30 (11.5–140)
ICU stay (days)	1 (1–3)	0 (0–0.5)	0 (0–0)
Hospital stay (days)	7 (5–8)	3 (3–4)	1 (0–4)
Any complication	19.2%	11.5%	8.2%
Type of complications, n			
Hemorrhage	3	0	0
Ureter injury	2	0	0
Organ injury	2	1	0
Infection	9	2	0
Ileus	1	0	0
Other	23	2	2

Similarly, postoperative intensive care and hospital stay were more pronounced in patients undergoing radical hysterectomy, with a median ICU stay of 1 day (IQR 1–3) and a median hospital stay of 7 days (IQR 5–8), compared with 0 days and 3 days (IQR 3–4) for non-radical hysterectomy, and minimal stays following fertility-sparing procedures. Postoperative complications were most frequent after radical hysterectomy (19.2%), followed by non-radical hysterectomy (11.5%) and fertility-sparing procedures (8.2%). The type of postoperative complications differed between surgical procedures. Following radical hysterectomy, complications were most commonly classified as “other” (n = 23), followed by infections (n = 9), hemorrhage (n = 3), organ injury (n = 2), ureteral injury (n = 2), and ileus (n = 1). In contrast, complications after non-radical hysterectomy were infrequent and mainly included infections (n = 2), organ injury (n = 1), and other complications (n = 2). Fertility-sparing procedures were associated with the lowest complication burden, with only isolated cases of hemorrhage (n = 1) and other minor complications (n = 2).

No significant difference in postoperative complication rates was observed between laparoscopic and open surgery (χ² = 0.72, df = 1, p = 0.40).

### Evolution of surgical techniques over time

A marked temporal variation in the use of different surgical techniques was observed. LRH was the most frequently performed procedure overall (n = 98) and predominated during the earlier years of the study. Annual LRH case numbers were highest between 2014 and 2018, ranging from 13 to 15 procedures per year, followed by a pronounced decline from 2019 onwards, with only sporadic cases documented thereafter. Similarly, VALRH was mainly performed between 2014 and 2018, with peak numbers in 2016 (n = 8) and 2018 (n = 9). LAVRH was used infrequently throughout the study period and did not show a consistent temporal pattern. In contrast, the use of ARH increased over time. While only 1–4 ARH procedures per year were performed between 2014 and 2017, the annual number rose notably from 2018 onwards, reaching 10–14 cases per year between 2018 and 2023, making ARH the predominant radical approach in later years. TMMR was introduced later during the study period and was predominantly performed between 2019 and 2021, with no procedures recorded thereafter.

Non-radical hysterectomy techniques, including TLH and LAVH, were applied consistently throughout the study period. TLH showed a gradual increase over time, with higher annual case numbers observed in the later years, peaking in 2023 (n = 10). TLH accounted for 12.2% of procedures performed between June 2014 and June 2023 and increased to 47.6% between June 2023 and June 2024. Although this increase reached statistical significance (p < 0.001), the number of patients treated during the latter period was limited and the findings should therefore be interpreted with caution.

LAVH was performed regularly at low but stable frequencies without a clear temporal trend. Abdominal simple hysterectomy was rarely used and documented only in isolated cases.

Fertility-sparing procedures were performed throughout the entire observation period. Cervical conization was applied consistently across all years, with annual case numbers ranging from 2 to 7 procedures, whereas trachelectomy showed variable use without a distinct temporal trend.

### Temporal trends in laparoscopic versus open surgical techniques following publication of the LACC trial (2018)

**Figure 2:**
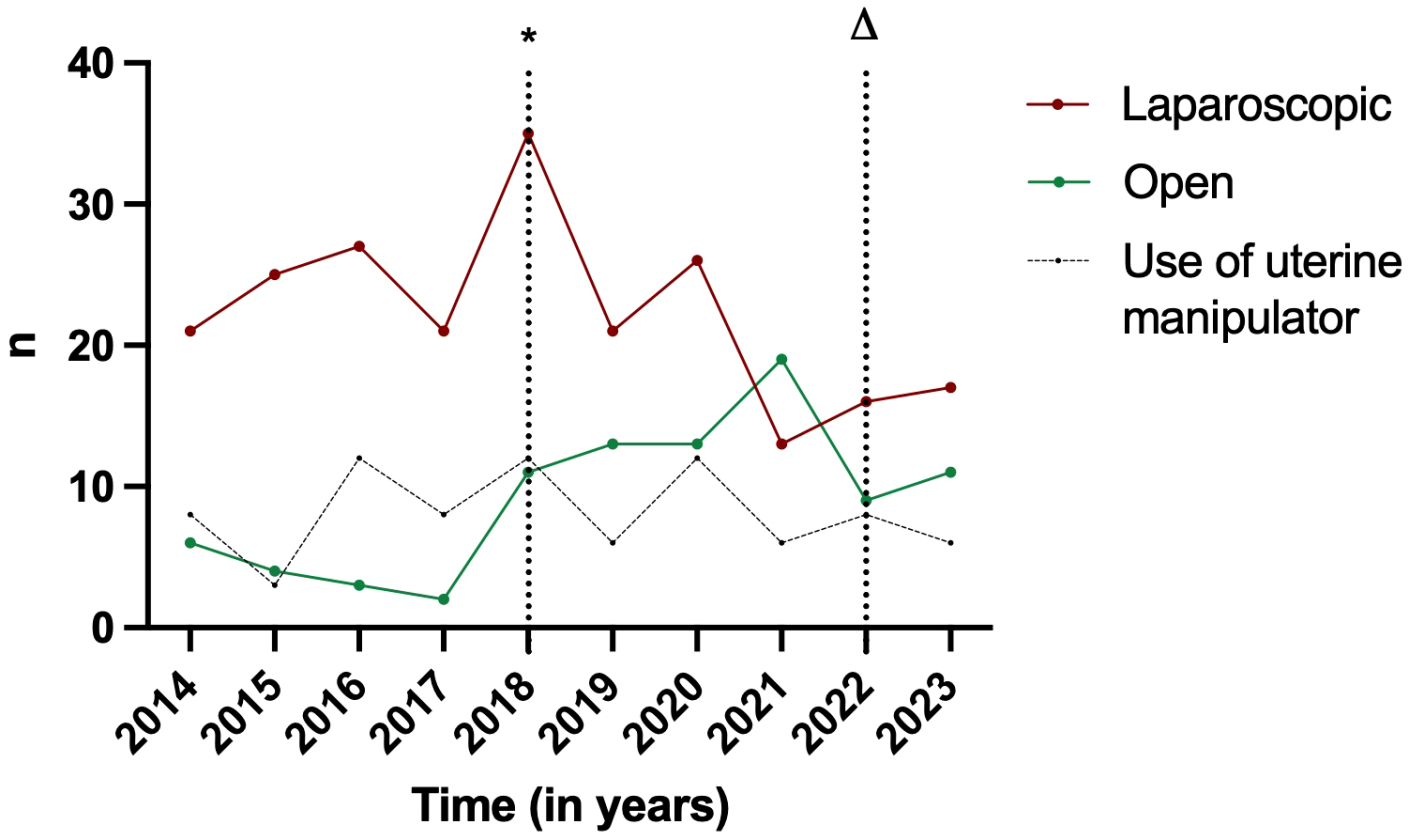
Temporal trends in laparoscopic versus open surgical techniques. *Publication of LACC trail, Δ guideline incorporation of LACC trial.

When surgical approaches were clustered according to surgical access (laparoscopic versus open), a clear temporal variation was observed over the study period (**Table 4**). Between 2014 and 2018, laparoscopic techniques predominated, accounting for 63.6–68.6% of procedures per year. During this early phase, open techniques were used less frequently, ranging from 6.5% to 21.6% annually. From 2019 onwards, a gradual shift in surgical access was noted. The proportion of laparoscopic procedures decreased to 56.8% in 2019 and 53.1% in 2020, followed by a more pronounced reduction in 2021, when laparoscopic techniques accounted for only 30.2% of all procedures. In parallel, the use of open techniques increased, reaching 35.1% in 2019, 26.5% in 2020, and peaking at 44.2% in 2021. In the subsequent years, the distribution between laparoscopic and open approaches appeared more balanced. Laparoscopic techniques accounted for 55.2% in 2022 and 50.0% in 2023, while open procedures represented 31.0% and 32.4%, respectively (**Figure 2**). In 2024, laparoscopic approaches accounted for 60.0% of cases and open approaches for 40%; however, this finding should be interpreted with caution, as data for 2024 were incomplete at the time of analysis, leading to a comparatively small number of recorded procedures.

**Table 4:** Surgical management before and after publication of the LACC trial.

Surgical procedure	Pre-LACC (2014–2018) n (%)	Post-LACC (2019–2024) n (%)
Laparoscopic techniques		
Laparoscopic radical hysterectomy (LRH)	68 (35.1)	30 (15.6)
Vaginally assisted laparoscopic radical hysterectomy (VALRH)	29 (14.9)	14 (7.3)
Laparoscopic-assisted vaginal radical hysterectomy (LAVRH)	5 (2.6)	3 (1.6)
Total laparoscopic hysterectomy (TLH)	15 (7.7)	31 (16.1)
Laparoscopic-assisted vaginal hysterectomy (LAVH)	12 (6.2)	18 (9.4)
*Total laparoscopic procedures*	*129 (66.5)*	*96 (50.0)*
Open techniques		
Abdominal radical hysterectomy (ARH)	21 (10.8)	53 (27.6)
Abdominal simple hysterectomy	1 (0.5)	1 (0.5)
Mesometrial resection (TMMR)	4 (2.1)	13 (6.8)
*Total open procedures*	*26 (13.4)*	*67 (34.9)*
Fertility-sparing surgery		
Cervical conization	22 (11.3)	22 (11.5)
Trachelectomy	17 (8.8)	12 (6.3)

When comparing surgical access between the two time periods (2014–2018 and 2019-2024), a statistically significant shift from laparoscopic to open techniques was observed (χ² = 21.36, df = 1, p < 0.001).

The use of a uterine manipulator varied over the study period and was documented in 84 of 391 procedures (21.5%). Across individual years, manipulator use ranged from 3 to 12 cases per year, without a consistent increasing or decreasing trend (**Figure 2**). Most procedures (303 cases, 77.5%) were performed without the use of a uterine manipulator. When clustered by time, uterine manipulators were used in 44 procedures between 2014 and 2018 and 38 procedures between 2019 and 2023, while procedures without manipulator use accounted for 147 and 153 cases, respectively (**Figure 2**). Data from 2024 reflects an incomplete observation year. No statistically significant difference in uterine manipulator use was observed between 2014–2018 and 2019–2023 (χ² = 0.39, df = 1, p = 0.53).

### Surgical access before and after guideline incorporation of the LACC trial (2022)

When surgical approaches were re-analyzed according to the period before (2014–2021) and after (2022–2023) incorporation of the LACC trial into national clinical guidelines, no statistically significant difference in the distribution of laparoscopic versus open techniques was observed. In the pre-guideline period, 189 laparoscopic and 71 open procedures were performed, compared with 33 laparoscopic and 20 open procedures after guideline implementation (Fisher’s exact test, p = 0.14). Although a numerical increase in open procedures was observed after 2022, this difference did not reach statistical significance.

The median age of women who underwent laparoscopic surgery was 45.5 years (IQR 37.3 - 61.0). Pathological tumor stages were predominantly microinvasive or small-volume disease, with pT1a1 (n = 13) and pT1b1 (n = 12) representing the most frequent stages. Only one patient presented with pT1b2 disease. All patients were either pN0 (n = 25) or pNx (n = 9), and no nodal-positive cases were treated laparoscopically in this period. Histologically, most tumors were squamous cell carcinomas (71%), followed by adenocarcinomas (26%). When analyzing tumor stage distribution in patients treated after guideline implementation (2022–2023), no significant association between pathological tumor stage and surgical access (laparoscopic vs. open) was observed (χ² = 0.00, p = 1.00). Microinvasive and ≥pT1b tumors were evenly distributed between both surgical approaches.

### Age-stratified surgical management

When stratified by age, differences in the distribution of surgical techniques were observed. In patients younger than 65 years, LRH was the most frequently performed procedure (24.1%), followed by ARH (17.6%), VALRH (11.0%), and TLH (12.5%). Fertility-sparing procedures, including cervical conization (12.5%) and trachelectomy (8.2%), were exclusively performed in this age group. In patients aged 65 years or older, LRH (34.2%) and ARH (31.6%) were the predominant surgical approaches, whereas fertility-sparing procedures were not performed. A significantly higher proportion of older patients underwent abdominal radical hysterectomy compared with younger patients (p = 0.048). Conversely, cervical conization was significantly more frequent in patients younger than 65 years (p = 0.013). No statistically significant age-related differences were observed for other surgical techniques (**Table 5**).

**Table 5:** Surgical procedures stratified by age group.

Surgical procedure	< 65 years %	≥ 65 years %	p-value*
Abdominal simple hysterectomy	0.3	2.6	0.19
Laparoscopic radical hysterectomy (LRH)	24.1	34.2	0.17
Laparoscopic-assisted vaginal radical hysterectomy (LAVRH)	2.0	2.6	0.56
Vaginally assisted laparoscopic radical hysterectomy (VALRH)	11.0	0.5	1.00
**Abdominal radical hysterectomy (ARH)**	17.6	31.6	0.048*
Vaginal hysterectomy (VH)	0	0	1.00
Laparoscopic-assisted vaginal hysterectomy (LAVH)	7.4	10.5	0.52
Total mesometrial resection (TMMR)	4.5	2.6	1.00
**Cervical conization**	12.5	0	0.013*
Trachelectomy	8.2	0	0.096
Total laparoscopic hysterectomy (TLH)	12.5	5.3	0.29

* means statistically significant (p < 0.05).

Bold values indicate statistically significant results (p < 0.05).

### Adjuvant therapy

Most patients were managed with tumor-specific follow-up, with no need for adjuvant treatment (349 patients, 89.3%). Adjuvant chemoradiotherapy was administered in 25 patients (6.4%), while adjuvant chemotherapy alone was given in 3 patients (0.8%). Other adjuvant treatment modalities were documented in 8 patients (2.1%). Information on adjuvant therapy was unavailable in 6 patients (1.5%).

### Recurrence and survival

Follow-up data were available for 388 of 391 patients. The median follow-up duration was 46.6 months (IQR 16.1–68.7; range 0.6–127.3) in the overall cohort. Patients diagnosed between 2014 and 2018 had a median follow-up of 68.7 months (IQR 37.3–84.2), whereas those diagnosed between 2019 and 2024 had a median follow-up of 28.6 months (IQR 8.5–49.2).

During follow-up, disease recurrence was documented in 22 patients (5.6%). The most frequent site of recurrence was the vaginal cuff, observed in 10 patients (2.6%). Pelvic lymph node recurrence occurred in 2 patients (0.5%), while no paraaortic lymph node recurrences were observed. Distant metastases were documented in 8 patients (2.1%). Among the 22 patients with documented recurrence, 11 had undergone minimally invasive surgery, 9 open surgery, and 2 fertility-sparing procedures. Detailed recurrence rates according to surgical procedure are shown in **Table 6**. Recurrence was observed in 8 of 98 patients (8.2%) after LRH, 2 of 43 patients (4.7%) after VALRH, 2 of 46 patients (4.3%) after TLH, and 8 of 74 patients (10.8%) after abdominal radical hysterectomy. Two recurrences occurred after fertility-sparing procedures (one after conization and one after trachelectomy). These findings should be interpreted descriptively, given the limited number of recurrence events and differences in baseline patient characteristics between surgical groups. Among vaginal cuff recurrences, 6 occurred after minimally invasive and 3 after open surgery.

**Table 6:** Recurrence according to surgical procedure.

Surgical procedure	Recurrences/total	Recurrence rate (%)
Laparoscopic radical hysterectomy (LRH)	8/98	8.2
Vaginally assisted laparoscopic radical hysterectomy (VALRH)	2/43	4.7
Total laparoscopic hysterectomy (TLH)	2/46	4.3
Abdominal radical hysterectomy (ARH)	8/74	10.8
Fertility-sparing procedures*	2/73	2.7

*One recurrence occurred after cervical conization and one after trachelectomy.

To further address the heterogeneity of the study population, an exploratory subgroup analysis of recurrence rates according to FIGO stage was conducted. Patients with microinvasive disease (FIGO IA–IA2) demonstrated a markedly lower recurrence rate than patients with FIGO stage IB or higher (0.7% vs. 8.3%). Specifically, only one recurrence occurred among 138 patients with microinvasive disease, whereas 21 recurrences were observed among 253 patients with FIGO stage IB or higher.

Among patients who developed recurrence, pathological tumor stages were predominantly pT1b disease, including 16 patients (72.7%) with pT1b1 and 4 patients (18.2%) with pT1b2, whereas only two patients (9.1%) presented with microinvasive tumors. Most recurrent tumors were squamous cell carcinomas (63.6%), and the majority were graded as G2 (68.2%) or G3 (27.3%).

Recurrence was significantly associated with adverse pathological features. Patients with lymphatic invasion (L1) experienced recurrence significantly more often than those without lymphatic invasion (L0) (p < 0.001). Similarly, vascular invasion (V1) was significantly associated with recurrence compared with V0 status (p = 0.002). In contrast, resection status (R0 vs. R1) was not significantly associated with recurrence (p = 0.51).

With regard to adjuvant therapy, 7 of 22 patients with recurrence (31.8%) had received adjuvant radiochemotherapy (RCT), and 1 patient (4.5%) had undergone adjuvant chemotherapy alone, while the majority (63.6%) had been managed with tumor-specific follow-up only. Overall, 7 of 25 patients (28.0%) treated with adjuvant RCT and 1 of 3 patients (33.3%) treated with adjuvant chemotherapy alone developed recurrence. RCT was administered in 25 patients, predominantly in cases with pT1b tumors and additional risk factors such as lymphovascular invasion. Both open and minimally invasive radical hysterectomy approaches were represented in this subgroup, without a clear predominance of surgical access.

At last follow-up, 18 patients (4.6%) were deceased. Mortality was significantly associated with lymphatic (L1) and vascular invasion (V1) (p < 0.001 and p = 0.001, respectively), whereas resection status was not significantly associated with death (p = 0.10). However, the cause of death could not be unequivocally attributed to cervical cancer.

## Discussion

This multicenter real-world analysis provides comprehensive insight into current surgical management patterns in patients with early-stage cervical cancer in Germany over a ten-year period. The study cohort reflects a relatively young patient population (mean age at diagnosis 44 years) which is younger than in national epidemiological studies (mean 53 years) (2) but in accordance with international collectives (mean 44–46 years) (6, 7, 9). Due to the study design more than half of tumors were staged as pT1b1, whereas approximately one-third represented pT1a tumors. These disease stages correspond to the clinical scenarios most affected by ongoing debate regarding the optimal extent and approach of surgery following the results of the LACC and SHAPE trial (10). The relatively high proportion of microinvasive tumors (pT1a1/pT1a2) may have contributed to the favorable oncologic outcomes observed in this cohort and limits direct comparability with the LACC trial population.

A major finding of this study is the pronounced temporal shift in surgical strategy, with laparoscopic techniques predominating before 2018. Following publication of the LACC trial, which demonstrated inferior disease-free and overall survival after minimally invasive surgery, a substantial decline in laparoscopic approaches was observed, accompanied by an increase in abdominal radical hysterectomy (5, 6). In parallel, national and international guidelines were updated to recommend an open surgical approach (4, 11, 12). Notably, laparoscopic approaches were still performed in 25.6% of cases after 2018. This observation is consistent with international data, which demonstrate a rapid but incomplete decline in the use of minimally invasive radical hysterectomy following the LACC trial, with a substantial proportion of patients still undergoing minimally invasive procedures in routine clinical practice (12–15). These findings suggest that, despite clear evidence favoring an open surgical approach, the implementation of guideline recommendations remains heterogeneous. The persistence of minimally invasive techniques may reflect ongoing clinical uncertainty, as well as the selective use of these approaches in patients with presumed low-risk disease. In addition, institutional practice patterns and surgeon experience are likely to contribute to this variability (16). The persistent use of minimally invasive approaches may partly reflect increasing selection of patients with low-risk and microinvasive tumors over time. When interpreting these temporal trends, it should be noted that the category of laparoscopic procedures included both radical and non-radical surgical approaches with different oncologic indications and therapeutic objectives. Consequently, changes in laparoscopic procedure rates over time may reflect not only changes in surgical access but also evolving concepts regarding the extent of surgery. The reasons underlying the continued use of minimally invasive surgery after publication of the LACC trial cannot be fully determined from our dataset. However, patients treated laparoscopically in the later study period predominantly presented with low-risk disease characteristics, including microinvasive tumors, early FIGO stages, and negative lymph node status. This suggests that MIS may have been preferentially reserved for carefully selected patients perceived to be at lower oncologic risk. Information regarding preoperative tumor size, imaging findings, surgeon preference, institutional policies, or multidisciplinary decision-making processes was not systematically available and therefore could not be evaluated in the present study.

Interestingly, when analyzing surgical practice before and after formal incorporation of the LACC trial into national clinical guidelines in 2022, no further statistically significant shift was observed. This suggests that the major change in surgical strategy had already occurred in the years following publication of the trial. The absence of an additional effect after guideline implementation may therefore reflect early evidence-driven adaptation within the gynecologic oncology community rather than delayed adherence to guideline updates. Taken together, these findings suggest that publication of the LACC trial represented an important catalyst for practice change, while evolving guideline recommendations, increasing patient selection, institutional experience, and broader developments in cervical cancer surgery likely also contributed to this transition. The observed change in surgical practice is consistent with international data indicating a global shift towards open radical hysterectomy for early-stage cervical cancer following the LACC trial, as several studies have confirmed inferior oncologic outcomes of minimally invasive surgery (17, 18).

A number of tumor-related and technical factors may influence these outcomes. These include tumor size (<2 cm vs. ≥2 cm) (17, 19) lymph node involvement, resection margins, and surgical technique, particularly the use of a uterine manipulator. In the present cohort, a uterine manipulator was used in 21.5% of cases. However, detailed information regarding the specific type of uterine manipulator and technical aspects of its application was not consistently available in this retrospective cohort. Therefore, a more detailed analysis of potential associations between manipulator characteristics and recurrence patterns could not be performed. Nevertheless, increasing evidence suggests that technical aspects of minimally invasive surgery, including uterine manipulation and intracorporeal colpotomy, may contribute to tumor dissemination and adverse oncologic outcomes (20, 21). In contrast, a limited number of studies have reported comparable oncologic outcomes between minimally invasive and open surgery, including similar recurrence rates and comparable 5-year disease-free and overall survival (22–24). Preoperative conization has been associated with a reduced risk of recurrence following minimally invasive surgery, particularly in tumors <2 cm (19, 25–27). This may partly explain why minimally invasive approaches are still applied in selected low-risk patients, despite the findings of the LACC trial. Interestingly, after the initial sharp decline of laparoscopic approaches in our cohort after 2018, a more balanced distribution between laparoscopic and open techniques re-emerged in subsequent years. A further major point of discussion in the management of early-stage cervical cancer arose following the publication of the SHAPE trial data, which demonstrated that, in low-risk cervical cancers (<2 cm, limited stromal invasion), simple hysterectomy was non-inferior to radical hysterectomy (7, 10). In our cohort simple hysterectomy (laparoscopic and open) posed a total of 24.4% of the cases, yet there is a notable increase in numbers over time with an increase especially in numbers of total laparoscopic hysterectomy (7.7% to 16.1%). In our cohort, the proportion of total laparoscopic hysterectomy increased from 12.2% between June 2014 and June 2023 to 47.6% between June 2023 and June 2024. In our cohort, the proportion of total laparoscopic hysterectomy increased from 12.2% between June 2014 and June 2023 to 47.6% between June 2023 and June 2024. However, the number of patients treated during the latter period was limited and the observation interval was short. Consequently, these findings should be regarded as exploratory and hypothesis-generating rather than definitive evidence of a change in surgical practice. Although the observed trend temporally coincided with the initial presentation of the SHAPE trial results, a direct influence of the trial on clinical decision-making cannot be established from the present data. A change in fertility-sparing approaches such as conization and trachelectomy was not seen in our cohort and numbers remained unchanged pre and post LACC publication (conization 11%, trachelectomy 6-8%).

The age-stratified analysis revealed expected treatment differences. Of course, fertility-sparing procedures (conization and trachelectomy) were exclusively performed in younger women, whereas older patients (<65 years) more frequently underwent abdominal radical hysterectomy 31.6% (*p-value 0.048*). Regarding other surgical approaches no significant differences where to be found. The literature suggests that older women (>65 years) participate less frequently in cervical cancer screening programs and are therefore more often diagnosed at more advanced tumor stages. In addition, clinical decision-making in this population frequently deviates from guideline recommendations. Consequently, worse progression-free and overall survival have been reported for these patients (28–30). These findings cannot be confirmed or refuted based on our cohort, as the study design resulted in a study population predominantly composed of early-stage tumors. However, it is noteworthy that abdominal surgical approaches were used more frequently in this age group. In this context, it remains a matter of discussion whether this was due to a higher burden of comorbidities, rendering laparoscopic procedures riskier, shorter surgical times within open surgery, or whether it reflected adherence to current guideline recommendations.

Overall, perioperative outcomes in this cohort were favorable; however, these findings should be interpreted in the context of the heterogeneous surgical procedures included. Complication rates were low, with no complications reported in 84.4% of patients and urinary complications occurring in only 0.5% of cases. Most patients did not require intensive care support (79.3%). The low reported complication rates should be interpreted with caution, as retrospective multicenter data collection may underestimate perioperative morbidity, particularly in the absence of prospective monitoring and standardized reporting. Importantly, perioperative outcomes differed substantially according to the type of surgical procedure. Radical hysterectomy was associated with longer operative times (median 233 minutes, IQR 157–335), longer postoperative hospital stays (median 7 days, IQR 5–8), and higher complication rates (19.2%) compared with non-radical hysterectomy (97 minutes, IQR 81–154; 3 days, IQR 3–4; 11.5%) and fertility-sparing procedures (30 minutes, IQR 11.5–140; 1 day, IQR 0–4; 8.2%). These findings highlight that overall favorable perioperative outcomes are partly driven by the inclusion of less invasive procedures and should therefore be interpreted with caution. These outcomes suggest high procedural safety across centers and reinforce the feasibility of both open and minimally invasive techniques in experienced hands. No significant difference in postoperative complication rates was observed between laparoscopic and open surgery (p = 0.40). Completion surgery was rarely required, indicating adequate primary surgical planning and staging accuracy.

The findings of the subgroup analysis further highlight the importance of stage distribution when interpreting the oncologic outcomes of the present study. More than one-third of the cohort consisted of patients with microinvasive disease (FIGO IA–IA2), a population that was largely underrepresented in the LACC trial. While only one recurrence was observed among 138 patients with microinvasive disease (0.7%), the recurrence rate among patients with FIGO stage IB or higher was substantially higher (8.3%). These findings suggest that the favorable overall recurrence and survival outcomes observed in our cohort may, at least in part, be attributable to the substantial proportion of low-risk tumors. Consequently, caution is warranted when comparing our results with studies including a higher proportion of patients with larger-volume early-stage cervical cancer. Recurrence occurred in only 5.9% of patients, most commonly at the vaginal cuff. Pelvic lymph node recurrence was rare, and distant metastases were observed in only 2.1% of patients. 18 patients had died at last follow-up, and deaths could not be unequivocally attributed to cervical cancer. Although survival analyses were not the primary endpoint of this descriptive study, these findings support the overall effectiveness of current surgical management strategies in early-stage cervical cancer and are in line with historically high survival rates reported for this disease stage (5, 19, 31). In our cohort, recurrence was associated with adverse pathological features such as lymphatic and vascular invasion. Previous studies have also highlighted the potential relevance of surgical technique in cervical cancer surgery. For example, the SUCCOR study reported inferior oncologic outcomes after minimally invasive surgery compared with open radical hysterectomy and identified uterine manipulator use as a potential factor associated with relapse risk (26). In our recurrence cohort, vaginal cuff recurrences were more frequently observed after minimally invasive procedures. However, manipulator use was inconsistently documented and detailed technical aspects such as intracorporeal colpotomy, protective vaginal closure, or the specific type of uterine manipulator were not reliably captured. Therefore, conclusions regarding surgical tumor hygiene or intraoperative tumor dissemination cannot be definitively drawn from the present dataset. These unresolved questions regarding surgical tumor hygiene and possible intraoperative tumor dissemination represent an important rationale for ongoing prospective studies, including the RACC (NCT03719547) and G-LACC (NCT06489795) trials, which are expected to provide further evidence regarding the oncologic safety of minimally invasive surgery in carefully selected patients. This study represents one of the few large-scale, multicenter real-world analyses capturing surgical trends over a full decade, thereby providing a robust picture of evolving practice patterns in Germany. The inclusion of detailed perioperative and oncological outcome data strengthens the clinical relevance of the findings. However, some limitations must be acknowledged. The retrospective design introduces inherent bias, and data completeness varied across variables, particularly regarding adjuvant therapy. Selection bias cannot be excluded, as surgical choice likely reflected institutional expertise and surgeon preference. Follow-up duration was variable and not standardized across centers, which may underestimate late recurrences or mortality. Due to the limited number of recurrence events (n = 22), deaths (n = 18), and major complications, multivariable regression analyses were not considered statistically robust and were therefore not performed. Consequently, outcome comparisons should be interpreted as descriptive and exploratory. Moreover, the study does not directly compare oncological outcomes between surgical approaches, and conclusions regarding oncological superiority must therefore be drawn cautiously. Furthermore, detailed intraoperative technical parameters potentially relevant to oncologic outcomes, including the type of uterine manipulator, intracorporeal colpotomy, and protective vaginal closure techniques, were not systematically documented across centers. Due to the limited number of recurrence events (n = 22) and deaths (n = 18), statistical power for outcome analyses was restricted. Consequently, the observed associations should be interpreted as exploratory, and multivariable analyses to identify independent predictors were not considered sufficiently robust. Although operative reports were reviewed where available, these variables were inconsistently reported and could therefore not be reliably extracted for the entire cohort.

In conclusion, this real-world multicenter study demonstrates substantial temporal changes in the surgical management of early-stage cervical cancer in Germany, including a marked shift from minimally invasive to open radical hysterectomy during a period that followed publication of the LACC trial and coincided with evolving guideline recommendations and changing treatment paradigms. Despite this reorientation, minimally invasive and fertility-sparing procedures continued to play an important role in selected patients. In addition, a recent increase in less radical minimally invasive approaches, including total laparoscopic hysterectomy, was observed after mid-2023, temporally coinciding with the initial presentation of the SHAPE trial results, although based on a limited number of cases. Overall, perioperative morbidity was low and oncological outcomes were favorable across the cohort.

## Data Availability

The raw data supporting the conclusions of this article will be made available by the authors, without undue reservation.
